# SYK expression in human breast cancer

**DOI:** 10.1186/1477-3163-4-7

**Published:** 2005-04-20

**Authors:** AE Elkak, W AL Sarakbi, K Mokbel

**Affiliations:** 1 The Breast Unit, St George's Hospital and Medical School, Blackshaw Road London, SW17 0QT, UK

## Abstract

**Background:**

Syk (Splenic Tyrosine Kinase) is an intracellular receptor protein kinase involved in cell proliferation, differentiation and phagocytosis. It has been studied in T and B lymphocytes, NK cells and platelets. The strong expression of Syk in mammary gland prompted research into its potential role in mammary carcinogenesis. There have been very few studies about its role in breast cancer with conflicting results. This study aims to investigate the hypothesis that Syk expression is down-regulated in breast cancer compared with ANCT and the association between its expression and clinicopathological parameters.

**Materials and methods:**

mRNA was extracted from 48 breast cancer specimens. Relative Syk to ribosomal RNA expression was determined by RT-PCR and Taqman methodology. Mann-Whitney U test was used to examine the association between Syk expression in cancer and ANCT. Spearman's rank correlation test was used to examine the association between Syk expression in tumours and patients' age, tumour size, tumour grade, estrogen and progesterone receptor status, lymph node metastasis, vascular invasion and clinical outcome.

**Results:**

The median for the relative value of Syk expression was 0.17 and 0.18 (range: 0.12 – 0.56 and 0.0 – 1.77) for tumours and ANCT respectively. There was no significant association between Syk expression in cancers and ANCT (p= 0.598) nor between Syk expression in tumours and patients' age, tumour size, tumour grade, estrogen and progesterone receptor status, lymph node metastasis, vascular invasion or prognosis.

**Conclusion:**

This study shows that Syk mRNA expression does not seem to vary between breast tumours and ANCT. Furthermore, we observed no significant association between Syk expression and clinicopathological parameters.

## Introduction

Breast cancer development and progression are thought to occur through a multistep process, including oncogene activation and mutation or loss of tumour suppresser genes [1]. Protein-tyrosine kinases (PTKs) have been classified into two families: trans-membrane receptors family and non- receptor family [2-5]. More than 30 receptor type PTKs have been identified in mammals and divided into 11 subfamiliies [6], of which we mention Tec, JAK, Sck, Fes, Abl, Fak, Scr and Syk [6,7]. Syk PTKs family comprises Syk and ZAP-70 [6]. Syk (named after Spleen Tyrosine Kinase) is associated with cell surface receptors to amplify receptor-activated signals inside the cells [6]. Syk family contains tandem Scr homology 2 (SH2) and c-terminal kinase domains interrupted by two interdomains A and B [8-12]. Syk assembles into signalling complexes at the plasma membrane via interaction between its tandem SH2 domains and a tyrosine-phosphorylated immunoreceptor tyrosine-based activation motif (ITAM), and this binding activates Syk [6,11,13,14] which is then tyrosine phosphorylated by autophosphorylation [5,6]. ITAM becomes phosphorylated on both tyrosine residues creating high affinity binding sites for the tandem SH2 domains [15]. Receptors containing these ITAMs act to initiate an activation cascade [16]. For example, stimulation of the B cell receptors (BCR) initiates a biochemical cascade, in which PTKs activation is the earliest known event [16]. Thus, Syk is recruited, activated by binding of SH2 to ITAM and then subsequently phosphorylated by autophosphorylation. Activated Syk phosphorylates its own substrate, which mediates further functions in the cells [16]. Syk is involved in mediating diverse cellular responses such as proliferation, differentiation and phagocytosis [12,17,18]. This includes T cell receptor (TCR) function and thymocyte development [7,12], B cell antigen receptor signalling [19] and natural killer (NK) cell differentiation (although not essential) and platelet activation by collagen [13]. Syk is not tyrosine phosphorylated in resting cells, possibly because it is dephosphorylated by specific protein tyrosine phosphatases [6].

Syk expression was found in a variety of organs such as spleen, heart, mammary gland [20] and in hepatocytes [21], haemtopoietic cells [22] and colonic carcinoma cells [23]. In an article published in Nature, Coopman et al [18] have reported that Syk was commonly expressed in normal human breast tissue, benign breast lesions and low-tumorigenic breast cancer cell lines whereas Syk mRNA and protein were low or undetectable in invasive breast carcinoma tissue and cell lines. In contrast with that, Fluck et al [20] demonstrated that aggressive metastasising mammary gland tumours expressed higher level of Syk compared with well differentiated non-metastasising tumours in a murine model of breast cancer. More recently, Yuan and colleagues [24] demonstrated that Syk was frequently inactivated through an epigenetic pathway in breast cancer. Using breast cancer cell lines, and human breast cancer tissues and PCR methodology, the authors observed that Syk was hypermethylated and therefore inactive in 12 (32%) of 37 breast tumours whereas all of the matched neighbouring normal breast tissue exhibited unmethylated DNA status (i.e. active Syk). Such observations are consistent with the hypothesis that Syk functions as a tumour suppresser gene and DNA methylation of this gene seems to play an important role in mammary carcinogenesis [25,26]. Other authors [22,27] have demonstrated that Syk is a prognostic factor in breast cancer. However, Wang et al, using immunoblotting technique, have found that breast cancer tissues expressed variable levels of Syk compared with normal breast tissues [5].

The present study aims to determine Syk mRNA expression in both breast cancer and adjacent normal breast tissue (ANCT) using RT-PCR methodology and correlate the expression in cancer with patients' age, tumour size, tumour grade, estrogen and progesterone receptor status, lymph node metastasis, vascular invasion and clinical outcome.

## Materials and methods

Institutional guidelines including ethical approval and informed consent were followed. Patients were treated with wide local excision or mastectomy and axillary node dissection. Patients with estrogen and /or progesterone positive tumours received tamoxifen. Adjuvant radiotherapy was administered to all patients who had breast conserving surgery and to patients undergoing mastectomy for node positive disease. Chemotherapy was given to fit patients (<65 years) with lymph node involvement or high grade tumours.

A 50 mg tumour specimen and an other 50 mg of macroscopically normal adjacent normal breast tissue (ANCT) were obtained from each patient. They were frozen in liquid nitrogen within 30 minutes of excision and stored at -70°C until use.

### RNA extraction

mRNA was extracted from 25 tumours and matching 23 ANCT using Absolutely RNA, RT-PCR Miniprep (Stratagene) Kit according to the manufacturer's protocol. Extraction of RNA was carried out in a laboratory separate to that used for RT-PCR.

### RT Step

cDNA was synthesised. Conditions for reverse transcriptase were 25°C for 10 minutes, 48°C for 30 minutes and 95°C for 5 minutes. Genomic contamination was tested for and was virtually nil.

### PCR

Taqman RT-PCR was performed for each sample using ABI PRISM 7700 Sensor Detection System (Applied Biosystems). The primers were designed using the Primer Selection Software. The primers used were ATT AGC AGA AGC AAA TGT CAT GCA (forward) and CCT CGC ATA TCC CGA TCA TC (reverse). PCR was carried out for 40 cycles of 15 seconds at 95°C and 1 minute at 60°C.

The relative expression of Syk to that of ribosomal RNA was determined using the Sequence Detection System Software. This experiment was carried out three times and the average values were calculated and used for statistical analysis. The positive and negative controls were ribosomal RNA and sterile water respectively.

### Statistical analysis

Mann-Whitney U test was used to examine the association between Syk expression in cancer and ANCT. Spearman's rank correlation test was used to examine the association between Syk expression in tumours and patients' age, tumour size, tumour grade, estrogen and progesterone receptor status, lymph node metastasis, vascular invasion and clinical outcome.

## Results

25 breast tumours and matching 23 ANCT were included in this study with patients age range 25–75 years (median 56.5, mean 56.80).

### Syk expression in tumours and ANCT

**Figure 1:**
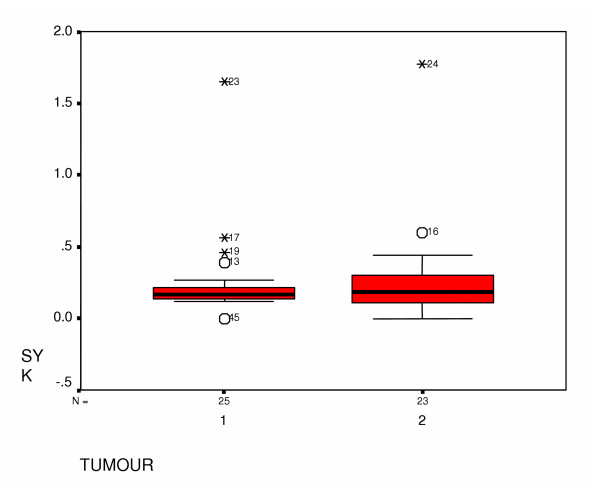
Scatter plot to show the relationship between Syk expression in cancers and normal breast tissues.

**Figure 2:**
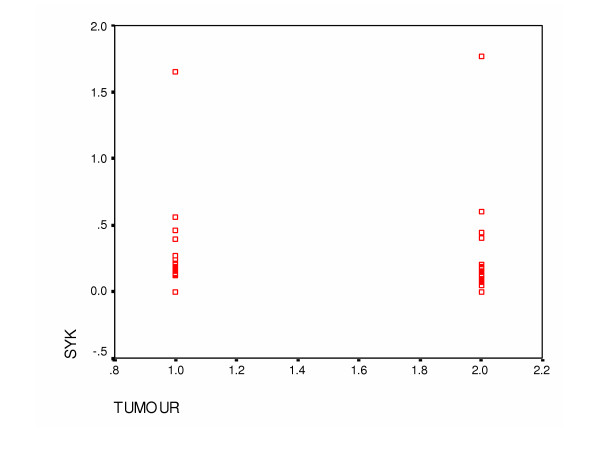
Box plot to show the relationship between Syk expression in cancers and normal breast tissues.

The median for the relative value of Syk expression was 0.17 and 0.18 (range: 0.12 – 0.56 and 0.0 – 1.77) for tumours and ANCT respectively. Mann-Whitney U test showed no significant association between Syk expression in cancers and ANCT (p= 0.598). (Figures 1 and 2)

### Correlation between Syk expression in tumours and clinicopathological parameters

The expression of Syk in tumours was correlated with patients' age, tumour size, tumour grade (well, moderately or poorly differntiated), estrogen and progesterone receptor status (positive, weak positive or negative), lymph node metastasis, vascular invasion, recurrence and clinical outcome (distant metastasis and death with a median follow up of 1.95 years). Spearman's rank correlation test showed no significant association between Syk expression in tumours and patients' age, tumour size, tumour grade, estrogen and progesterone receptor status, lymph node metastasis, vascular invasion or prognosis survival curves. Table 1 demonstrates the correlation coefficient and p values for the variables studied.

**Table 1:** the correlation coefficient and p values for the variables studied.

	Age	Size	Hist	Grade	LN	Vasc	ER	PR	Rec	OC
Corr coef	-0.104	0.102	0.01	0.137	-0.007	0.141	0.089	-0.294	0.130	0.339
P value	0.653	0.679	0.963	0.626	0.977	0.603	0.708	0.208	0.595	0.143

## Discussion

Our report in the present article represents the largest study of Syk expression in human breast cancer and ANCT. It has that Syk mRNA is expressed in both human breast cancer and ANCT specimens. This observation is consistent with the established role of Syk in the control of cell proliferation and intracellular signalling in epithelial cells and immunocytes [12,17,18]. The lack of statistically significant difference between Syk mRNA expression in the whole tumour and ANCT specimens observed in this study may represent a true observation and therefore, Syk should not be considered a tumour suppressor gene. It does not, however, exclude the presence of different levels of Syk expression in malignant and benign mammary epithelial cells. Breast tumours are heterogenous lesions [5] that contain various components including fibroblasts and lymphocytes. The latter can make up to 45% of the whole breast tumour volume [28]. Lymphocytes are known to express significant levels of Syk and this could lead to a false overestimation of Syk mRNA in epithelial cancer cells. A more accurate estimation of Syk mRNA in epithelial elements can be achieved by using Laser capture microdissection (LCM) in order to separate epithelial cells from other components in both tumours and ANCT. Alternatively, in situ hybridization and immunohistochemistry techniques can be used to identify the cellular source of the enzyme (Coopman et al). The ANCT in our study was obtained from macroscopically normal breast tissue more than 10 mm away from the primary tumour. However, one cannot exclude the presence of malignant epithelial cells in these specimens, as histopathological verification of ANCT was not carried out. Another limitation of the present study is the fact that we measured mRNA transcript rather than the protein, which is the effective end product of the gene. However, experimental data from breast cancer cell lines suggest that there is a good correlation between mRNA and protein expression (Coopman et al). Furthermore, the presence of splice variants of Syk mRNA (Fluck et al) represents another limitation to our RT-PCR methodology. Our results also show that Syk mRNA expression in breast tumours as determined by RT-PCR methodology is independent of tumour stage and therefore it is unlikely to play a prognostic role in patients with breast cancer.

## Abbreviations

Corr Coef: Correlation coefficient

Hist: Histology

LN: Lymph node metastasis

Vasc: Vascular invasion

ER: Estrogen receptors

PR: Progesterone receptors

Rec: Recurrence

OC: Clinical outcome
